# Genotypes and haplotypes in the insulin-like growth factors, their receptors and binding proteins in relation to plasma metabolic levels and mammographic density

**DOI:** 10.1186/1755-8794-3-9

**Published:** 2010-03-19

**Authors:** Margarethe Biong, Inger T Gram, Ilene Brill, Fredrik Johansen, Hiroko K Solvang, Grethe IG Alnaes, Toril Fagerheim, Yngve Bremnes, Stephen J Chanock, Laurie Burdett, Meredith Yeager, Giske Ursin, Vessela N Kristensen

**Affiliations:** 1 Department of Genetics, Institute for Cancer Research, Oslo University Hospital Radiumhospitalet, Montebello 0310, Oslo, Norway; 2 Institute of Community Medicine, University of Tromsø, Breivika, Norway; 3 University of Alabama at Birmingham, School of Public Health, Department of Epidemiology, Birmingham, Alabama, USA; 4 Department of Biostatistics, Institute of Basic Medical Science, University of Oslo, Oslo, Norway; 5 Department of Genetics, University Hospital of North Norway, Tromsø; Norway; 6 NCI, NIH, Pediatric Branch, Bethesda, USA; 7 Core Genotyping Facility, Advanced Technology Center, National Cancer Institute, Bethesda, MD, 20892, USA; 8 Department of Nutrition, Institute of Basic Medical Sciences, University of Oslo, Oslo, Norway; 9 Department of Preventive Medicine University of Southern California Keck School of Medicine, Los Angeles, CA, USA; 10 Faculty of Medicine, (Faculty Division Ahus), UiO, Oslo, Norway

## Abstract

**Background:**

Increased mammographic density is one of the strongest independent risk factors for breast cancer. It is believed that one third of breast cancers are derived from breasts with more than 50% density. Mammographic density is affected by age, BMI, parity, and genetic predisposition. It is also greatly influenced by hormonal and growth factor changes in a woman's life cycle, spanning from puberty through adult to menopause. Genetic variations in genes coding for hormones and growth factors involved in development of the breast are therefore of great interest. The associations between genetic polymorphisms in genes from the IGF pathway on mammographic density and circulating levels of IGF1, its binding protein IGFBP3, and their ratio in postmenopausal women are reported here.

**Methods:**

Samples from 964 postmenopausal Norwegian women aged 55-71 years were collected as a part of the Tromsø Mammography and Breast Cancer Study. All samples were genotyped for 25 SNPs in IGF1, IGF2, IGF1R, IGF2R, IGFALS and IGFBP3 using Taqman (ABI). The main statistical analyses were conducted with the PROC HAPLOTYPE procedure within SAS/GENETICS™ (SAS 9.1.3).

**Results:**

The haplotype analysis revealed six haploblocks within the studied genes. Of those, four had significant associations with circulating levels of IGF1 or IGFBP3 and/or mammographic density. One haplotype variant in the IGF1 gene was found to be associated with mammographic density. Within the IGF2 gene one haplotype variant was associated with levels of both IGF1 and IGFBP3. Two haplotype variants in the IGF2R were associated with the level of IGF1. Both variants of the IGFBP3 haplotype were associated with the IGFBP3 level and indicate regulation in cis.

**Conclusion:**

Polymorphisms within the IGF1 gene and related genes were associated with plasma levels of IGF1, IGFBP3 and mammographic density in this study of postmenopausal women.

## Background

Increased mammographic density is one of the strongest independent risk factors for breast cancer [1-8]. The risk of developing breast cancer is four to six times higher in women with dense breast tissue compared to women with less dense tissue[2]. It has been estimated that breasts with more than 50% mammographic density give rise to one third of breast cancer cases[2]. Mammographic density is thus a stronger cancer risk factor than the most traditional risk factors such as nulliparity[1], age at first birth, age at menarche, age at menopause, use of postmenopausal hormone therapy (HT) and alcohol consumption. Mammographic density is influenced by age[2], body mass index (BMI)[2], parity[2], menopause status[2], HT[1,9], IGF1[10,11] and genetics[2]. Exposure to endogenous and exogenous steroid hormones and growth factors has been linked to both increased mammographic density and breast cancer risk.

It has been estimated from twin studies that as much as 65% of the variation in mammographic density may be due to hereditary factors[12,13]. The genetic factors that influence mammographic density might be the same as the ones involved in the development of breast cancer[14,15]. The identification of the genes coding for these factors may therefore provide a better understanding of the genetics and the biology of the breast. Of particular interest are the insulin-like growth factor 1 and 2 (*IGF1/2*), which both have the ability to stimulate cell proliferation and inhibit cell death in many tissue types[16]. IGF1 is a mitogen predicted to be involved in the development of several human cancers, including breast cancer. In addition some studies have shown an association between circulating levels of IGF1 and increased levels of mammographic density [17-21]. In the bloodstream IGF1 binds to several IGF binding proteins (IGFBPs) which prolong its half-life and contribute to its delivery to target tissues[22]. IGFBP3, the principal carrier of IGF1 and IGF2[23], is mainly regulated by growth hormone (GH). IGFBP3 itself has the ability to promote apoptosis[24], thus it is sometimes referred to as an anticancer protein. The levels of IGF1 and IGFBP3 combined may be associated with breast cancer by stimulating proliferation of breast epithelial cells[22]. IGF1 bound to IGFBP3, may bind an acid-labile subunit (ALS) to form ternary complexes[25]. ALS is synthesized in the liver upon regulation of growth factors such as GH. By forming a ternary complex, ALS prolongs the half-lives of circulating IGFs from 10 minutes (free form), and 30-39 minutes (binary complex) to more than 12 hours. As a result, the reservoir of serum IGF1 levels in human adults can reach ~1000 fold that of insulin[26]. IGF1 and IGF2 both bind to the IGF1 receptor (IGF1R) to exert their growth promoting effects[27]. IGF2 may also bind to the IGF2 receptor (IGF2R) upon which it is internalized and degraded. Together, environmental and genetic factors determine the circulating levels of IGFs and their binding proteins[28]. Previous studies on polymorphisms/haplotypes in *IGFI, IGFBP3 *and *IGFALS *and their association to breast cancer susceptibility [25,29-31] and circulating levels of both IGFI[32] and IGFBP3[31] have been reported making them interesting targets in mammographic density studies. Tamimi *et al*. found positive correlation between common genetic variants in *IGF1 *and mammographic density[11]. Since variation in specific genes affects the levels of IGF1 and IGFBP3 and may influence mammographic density as well as breast cancer susceptibility, we set out to analyze 24 SNPs in *IGF1, IGF1R, IGF*2, *IGF2R, IGFBP3 *and *IGFALS *and their association with mammographic density and plasma levels of IGF1 and IGFBP3 among 964 postmenopausal women.

## Methods

### Study Population

The Tromsø Mammography and Breast Cancer Study [21,33-37] was conducted among postmenopausal women, ages 55 to 71 years, residing in the municipality of Tromsø, Norway, and attending the population-based Norwegian Breast Cancer Screening Program at the University Hospital of North Norway. The women were recruited in the spring of 2001 and 2002. After the women had undergone their mammographic screening, they were interviewed by a trained research nurse about reproductive and menstrual details, previous history of cancer, smoking status, and use of postmenopausal hormone therapy and other medications. The participants had their height measured to the nearest centimeter and their weight measured to the nearest half kilogram. The women had blood samples drawn and were given a questionnaire to be completed at home. The questionnaire elicited information on demographics, additional menstrual and reproductive factors, as well as lifestyle and dietary factors. All women signed an informed consent. The National Data Inspection Board and the Regional Committee for Medical Research Ethics approved the study. Altogether, 1,041 women were included in the study. This accounted for 70.1% of the women attending the Norwegian Breast Cancer Screening Program during the recruitment period[37].

We excluded 22 women because of a previously (*n *= 16) or newly (*n *= 6) diagnosed breast cancer and one woman because of ongoing chemotherapy treatment. Women who were 56 years or older or who reported no menstruation during the last 12 months, or whose serum follicle-stimulating hormone level was above 20 IU/L, were classified as postmenopausal. By these criteria, three women were equivocal for menopausal status and excluded. We further excluded 11 women whose mammograms were unreadable for technical reasons. Seventeen women with missing blood samples and 23 women due to missing SNP analysis values were also excluded, leaving 964 women for analysis. More details are described elsewhere. http://uit.no/density

### Mammographic classifications

Left craniocaudal mammograms were digitized using a Cobrascan CX-812 scanner (Radiographic Digital Imaging, Torrance, CA) at a resolution of 150 pixels/in. Percent and absolute mammographic densities were determined using the Madena computer-based threshold method, developed at the University of Southern California[38]. Briefly, the method was as follows: The digitized mammographic image was viewed on a computer screen. A reader (trained by GU) outlined the total area of the breast using a computerized tool, and the software then counted the number of pixels within the outline. Mammographic density was then assessed (by GU) by first identifying a region of interest that incorporated all dense areas except those representing the pectoralis muscle and other scanning artifacts, and then applying a yellow tint to all pixels within the region of interest shaded at or above a threshold intensity of gray. The software then counted the tinted pixels, which represent the area of absolute density (ABDEN). Percent density (PDEN) equals the amount of absolute density divided by the total breast area.

The reader of the mammograms did not have any information of the characteristics of the study participants. More details are given elsewhere[33].

### Peptide Assays

Nonfasting venous blood samples were obtained from the study participants at the day of mammographic screening. After centrifugation, plasma samples were stored at -70°C.

IGF1 and IGFBP3 levels were measured in ng/ml with the use of ELISA from Diagnostic Systems Laboratories, Inc. (Webster, TX). The IGF1 assays included an acid-ethanol precipitation to extract IGF1 from its binding proteins. Measurements were performed on previously never-thawed plasma samples.

All IGF1 and IGFBP3 assays were conducted at the laboratory for hormone analyses (Nutrition and Cancer Group, IARC, Lyon, France). The mean intrabatch coefficients of variation were 5.1% for IGF1 and 6.1% for IGFBP3. The interbatch coefficients of variation were 10.6% for IGF1 and 9% for IGFBP3.

The IGF1/IGFBP3 molar ratio was calculated as a possible indicator of IGF1 bioavailability. More details are described elsewhere[21].

### DNA extraction

Peripheral blood from healthy women was collected in EDTA-tubes. The DNA was isolated by phenol/chloroform extraction followed by ethanol precipitation using the Applied Biosystems Model 340A Nucleic Acid Extractor and stored in TE-buffer at 2-8°C. The sample concentrations were measured by UV/Vis spectrophotometer (Nanodrop ND-1000).

### Genotyping methods

5 ng of lyophilized sample DNA was used to perform a 5 ul Taqman (5' nuclease assay) reaction. Reactions were set up using 2.5 ul of the 2× Universal Master Mix (Applied Biosystems, Foster City, CA) and assay-specific concentrations of primers and probes. All reactions were set up in a 384 (96*4) well plate and heat-sealed using an ABgene ALPS 300 heat sealer and clear heat sealing film (ABgene, Rochester, NY). Reaction plates were thermocycled, and endpoint reads were conducted on the ABI 7900HT sequence detection system. Cluster Analysis was conducted on the scatter plot of Allele 1 Rn versus Allele 2 Rn. Genotypic segregation was displayed in the allelic plot, containing four distinct clusters, which represent the NTCs (no template controls) and three possible genotypes clusters along the horizontal, vertical and diagonal axes, which represent the Allele 1, Allele 2 and Allele 1/Allele 2 respectively. The data were exported in text format for further analysis. The sequences of the respective probes are given at http://snp500cancer.nci.nih.gov upon search for each SNP (rs number).

### Statistical analysis

Simple descriptive statistics and other analyses were performed on the final study population with the use of SAS^® ^9.1.3 software. For each SNP within the haplotype regions evaluated, we calculated the allele and genotype frequencies using programming algorithms written in Base SAS^®^.

The haplotype analyses were performed in SAS/GENETICS using the PROC HAPLOTYPE procedure. This procedure utilizes the Expectation Maximization (EM) algorithm to predict the maximum likelihood estimates of the haplotype frequencies assuming Hardy-Weinberg equilibrium. The standard errors and the confidence intervals are estimated, by default, under a binomial assumption for each haplotype frequency estimate. In addition, the linkage disequilibrium (LD) option in PROC HAPLOTYPE was specified which generated a likelihood ratio test for linkage disequilibrium testing a null hypothesis of no association between the SNPs within a given haplotype region. The null hypothesis was rejected for all haplotype regions evaluated in this paper; the haplotype frequency estimates from the alternative hypothesis are reported here. The haplotype frequency threshold was set to 0.05, and haplotypes with a lower frequency were not included in the subsequent haplotype-trend regression analysis done in SAS^®^.

We applied the programming algorithms for haplotype-trend regression as developed by SAS/GENETICS; these methods also utilize the SAS/STAT procedure PROC REG for the regression models[39,40]. Haplotype-trend regression models [41] were used to assess whether haplotypes were associated with absolute mammographic density and percent mammographic density in square centimeters, in addition to the levels of IGF1, IGFBP3 measured in ng/ml and their molar ratio IGF1/IGFBP3. Box-Cox transformations were applied to absolute mammographic density and percent density to more closely approximate a normal distribution in this study population [42]. The optimum power parameters of the Box-Cox transformation were obtained by the Dynamic programming called the Symplex method[43] and the code was written in R. All analyses were adjusted for age and BMI. The haplotypes within a gene were included individually in a model for comparison to the other haplotypes within the same gene [11].

For each haplotype region a model with haplotypes with frequencies >= 0.05 and the covariates age and BMI was compared to a model with age and BMI alone; the procedure computed the F statistic and the two-tailed significance probability for these models. In addition, all of the above-specified models were stratified by current HT users versus never and past HT users.

We also conducted additional analyses stratified by age and BMI and analyzed with general linear models using Box-Cox transformed values of mammographic density as the outcome.

We conducted further analyses of the association between individual SNPs in the haplotype regions and each of the outcome measurements. We assessed least square mean mammographic density by genotype adjusting for age and BMI using generalized linear models (PROC GLM). We estimated trend statistics by running separate models including genotype as an ordinal variable. For all the above-mentioned analyses we defined the results as statistically significant if the p-values were below 0.05.

## Results

### Characteristics of the Tromsø Mammographic and Breast Cancer Study

Shown in Table 1 are the selected characteristics of the participants. The median percent mammographic density was 9.6% (0.0-69.2%) with a mean of 12.7% (± 12.2%). The median absolute mammographic density was 14.8 cm^2 ^(0.0-155.2 cm^2^), with mean of 19.3 cm^2 ^(± 20.3 cm^2^).

**Table 1:** Selected characteristics of the 964 participants.

	All
**Mean**	
Age at screening, y	61.4 (± 4.6)
Age at menarche, y	13.3 (± 1.4)
Age at first birth*, y	22.9 (± 3.7)
Number of children*	2.9 (± 1.3)
Education, y	9.8 (± 3.4)
Age at menopause, y	48.6 (± 5.1)
BMI, kg/m^2^	27.3 (± 4.8)
**Frequency (%)**	
Ever Oral Contraceptive use	51.1
Parous	92.6
Ever postmenopausal hormone therapy use	43.4
Current postmenopausal hormone therapy use	26.0
Past postmenopausal hormone therapy use	17.3
Never postmenopausal hormone therapy use	56.6
Breast cancer in first degree relative	8.3
**Median**	
Percent mammographic density, %	9.6 (0-69.2)
Absolute mammographic density, cm^2^	14.8 (0-155.2)
Non-dense mammographic area, cm^2^	147.0 (21.6-448.9)
IGF1 (ng/ml)	28.7 (2.2-104.6)
IGFBP3 (ng/ml)	151.2 (52.17-241.5)

*Among parous women only.

Data collected as part of the Tromsø Mammography and Breast Cancer(TMBC) study in Tromsø, Norway in 2001-2002, presented as mean (± standard deviation), frequency (%), and median (range).

### Haplotype analysis

The allele and genotype frequencies for all studied SNPs in the genes IGF1, IGF1R, IGFBP3, IGF2, IGF2R and IGFALS are provided in Table 2.

**Table 2:** Allele and genotype frequencies of the 964 participants.

Gene		Gene ID	SNP ID	Allele 1 frequency Allele 2 frequency	A1/A1 N = (%) A1/A2 N = (%) A2/A2 N = (%)
IGF1	1	IGF1-02	rs6220	T = 0.66703 C = 0.33297	C/C = 104 (11.2) T/C = 410 (44.2) T/T = 414 (44.6)
	
	2	IGF1-04	rs2162679	G = 0.16772 A = 0.83228	G/G = 22 (2.3) G/A = 275 (28.9) A/A = 654 (68.8)

IGF2	1	IGF2-02	rs734351	T = 0.62393 C = 0.37607	C/C = 129 (13.8) T/C = 443 (47.5) T/T = 360 (38.6)
	
	2	IGF2-03	rs3213216	G = 0.61645 A = 0.38355	A/A = 131 (14.0) G/A = 456 (48.7) G/G = 349 (37.3)

IGF1R	1	IGF1R-05	rs2137680	G = 0.70768 A = 0.29232	A/A = 92 (9.7) G/A = 372 (39.1) G/G = 487 (51.2)
	
	2	IGF1R-18	rs2175795	G = 0.70952 A = 0.29048	A/A = 90 (9.5) G/A = 369 (39.0) G/G = 486 (51.4)
	
	3	IGF1R-06	rs907806	G = 0.09201 A = 0.90799	G/G = 15 (1.6) G/A = 145 (15.2) A/A = 791 (83.2)
	
	4	IGF1R-04	rs3743258	G = 0.72569 A = 0.27431	A/A = 71 (7.5) G/A = 377 (39.9) G/G = 498 (52.6)
	
	5	IGF1R-26	rs3743259	G = 0.30765 A = 0.69235	G/G = 86 (9.1) G/A = 407 (43.3) A/A = 448 (47.6)
	
	6	IGF1R-03	rs2272037	C = 0.57354 T = 0.42646	T/T = 171 (18.1) C/T = 464 (49.1) C/C = 310 (32.8)
	
	7	IGF1R-01	rs2229765	G = 0.56019 A = 0.43981	A/A = 184 (19.4) G/A = 465 (49.1) G/G = 298 (31.5)
	
	8	IGF1R-07	rs2016347	T = 0.52784 G = 0.47216	G/G = 202 (21.2) T/G = 495 (52.0) T/T = 255 (26.8)

IGF2R	1	IGF2R-05	rs1570070	A = 0.63097 G = 0.36903	G/G = 132 (14.0) A/G = 432 (45.8) A/A = 379 (40.2)
	
	2	IGF2R-01	rs894817	G = 0.68873 A = 0.31127	A/A = 97 (10.6) G/A = 375 (41.0) G/G = 442 (48.4)
	
	3	IGF2R-02	rs998075	G = 0.49840 A = 0.50160	G/G = 235 (25) G/A = 467 (49.7) A/A = 238 (25.3)
	
	4	IGF2R-11	rs998074	C = 0.50211 T = 0.49789	T/T = 235 (24.8) C/T = 474 (50) C/C = 239 (25.2)
	
	5	IGF2R-04	rs629849	G = 0.86456 A = 0.13544	A/A = 21 (2.2) G/A = 211 (22.6) G/G = 702 (75.2)
	
	6	IGF2R-07	rs2282140	C = 0.89504 T = 0.10496	T/T = 6 (0.6) C/T = 187 (19.7) C/C = 755 (79.6)
	
	7	IGF2R-03	rs1803989	T = 0.09746 C = 0.90254	T/T = 10 (1.1) T/C = 164 (17.4) C/C = 770 (81.6)

IGFALS	1	IGFALS-05	rs9282731	T = 0.00105 C = 0.99895	T/T = 0 (0) T/C = 2 (0.2) C/C = 946 (99.8)
	
	2	IGFALS-01	rs17559	T = 0.09057 C = 0.90943	T/T = 13 (1.4) T/C = 145 (15.4) C/C = 786 (83.3)
	
	3	IGFALS-02	rs3751893	T = 0.21186 C = 0.78814	T/T = 41 (4.3) T/C = 318 (33.7) C/C = 585 (62.0)

IGFBP3	1	IGFBP3-05	rs9282734	C = 0.00317 A = 0.99683	C/C = 0 (0) C/A = 6 (0.6) A/A = 941 (99.4)
	
	2	IGFBP3-04	rs2471551	G = 0.17766 C = 0.82234	G/G = 27 (2.9) G/C = 280 (29.8) C/C = 633 (67.3)

The estimated common haplotypes (frequency higher than 5%) for the 6 genes and their association to the parameters studied are shown in Table 3. Haplotypes in four of the six studied genes were found significantly associated with the plasma levels and/or mammographic density (Table 3). One haplotype variant TG in *IGF1 *was significantly associated with an increase in absolute mammographic density (p = 0.0334). However, upon stratification by current and past/never use of HT (See Additional files 1 and 2) this association was no longer significant (p = 0.1439 and p = 0.1271, respectively). In *IGF2 *the rare common haplotype variant CA was associated with increased levels of both IGF1 (p = 0.0014) and IGFBP3 (p = 0.0181), with significant global association for these parameters p = 0.0138, p = 0.0408, respectively. After stratification by HT, the association between this haplotype and IGF1 levels was still statistically significant (p = 0.0011), and the association with IGFBP3 levels was borderline statistically significant (p = 0.0730), for women currently taking HT. For women who were past or never users of HT the association was no longer significant (p = 0.1261 and 0.2194 respectively). Haplotypes 1 and 5 in the *IGF2 receptor *(*IGF2R*) were found associated with decreased levels of circulating IGF1, p = 0.0397 and 0.0455, respectively. In addition, the global p-value of 0.0524 indicated an overall borderline association of all seven listed haplotypes in *IGF2R *with the IGF1 level. Stratification by HT status also revealed a significant association between haplotype 1 and IGF1 levels in never/past HT users (p = 0.0353), but not in current users (p = 0.7907). On the other hand, the association between haplotype 5 and IGF1 levels was no longer statistically significant in never/past HT users (p = 0.2031) but borderline significant in current HT users (p = 0.0637).

**Table 3:** Associations of the common haplotypes with IGF1, IGFBP3 and mammographic density levels.

		IGF1 level	IGFBP3 level	IGFratio	PDEN^a^	ABDEN^b^
**IGF1**						
Haplotype	*Frequency*	p-value	p-value	p-value	p-value	p-value

1. CG	*0,1185*	0.5553	0.9493	0.6462	0.2337	0.2693
2. CA	*0,21486*	0.1424	0.5075	0.4549	0.5147	0.6701
3. TA	*0,61718*	0.7733	0.7103	0.9016	0.4486	0.8462
4. TG	*0,04947*	0.1683	0.7237	0.2713	0.1737	***0.0334***
Global association		0.3342	0.924	0.6641	0.3069	0.1167
						
**IGF2**						
Haplotype	*Frequency*	p-value	p-value	p-value	p-value	p-value

1. TG	*0,34537*	0.2769	0.9845	0.1648	0.2297	0.4051
2. CG	*0,27102*	0.6093	0.1440	0.3058	0.5776	0.7190
3. TA	*0,27821*	0.7660	0.9920	0.5860	0.5254	0.6165
4. CA	*0,10541*	***0.0014***	***0.0181***	0.0974	0.8518	0.9403
Global association		***0.0138***	***0.0408***	0.2712	0.6353	0.8313
						
**IGF1R**						
Haplotype	*Frequency*	p-value	p-value	p-value	p-value	p-value

1. GGAGATGT	*0,09532*	0.8216	0.6761	0.7512	0.8689	0.9008
2. GGAGATAG	*0,07847*	0.1245	0.0885	0.5697	0.1153	0.1678
3. GGAGACGT	*0,12829*	0.8324	0.9646	0.9343	0.4403	0.6585
4. GGAAGCAG	*0,05028*	0.4479	0.6971	0.4485	0.7200	0.8879
5. GGAGACAG	*0,10502*	0.8550	0.9531	0.8820	0.1567	0.2125
Global association		0.5384	0.5162	0.8893	0.3016	0.4638
						
**IGF2R**						
Haplotype	*Frequency*	p-value	p-value	p-value	p-value	p-value

1. GAATACC	*0,06242*	***0.0397***	0.1228	0.2853	0.2140	0.0784
2. AGGCGCC	*0,27961*	0.2376	0.3324	0.5912	0.7018	0.6786
3. GAGCGTC	*0,06251*	0.7378	0.8651	0.9925	0.9465	0.7463
4. GAATGCC	*0,11233*	0.4057	0.4787	0.9129	0.7414	0.5293
5. AGATGCC	*0,2451*	***0.0455***	0.0631	0.4429	0.7105	0.8569
6. AGATACC	*0,07038*	0.4484	0.3361	0.8486	0.9180	0.8191
7. GAGCGCT	*0,06327*	0.5430	0.4985	0.6552	0.0575	0.0622
Global association		0.0524	0.1619	0.8720	0.5036	0.2659
						
**IGFals**						
Haplotype	*Frequency*	p-value	p-value	p-value	p-value	p-value

1. CTC	*0,08951*	0.8055	0.0582	0.2993	0.4069	0.5648
2. CCC	*0,69765*	0.2290	0.6156	0.3095	0.8880	0.9099
3. CCT	*0,21171*	0.1412	0.0769	0.6082	0.6291	0.7605
Global association		0.3387	0.0523	0.4678	0.6624	0.8261
						
**IGFBP3**						
Haplotype	*Frequency*	p-value	p-value	p-value	p-value	p-value

1. AG	*0,17767*	0.1630	***0.0009***	0.1595	0.5689	0.2204
2. AC	*0,81917*	0.1635	***0.0008***	0.1531	0.5551	0.2302
Global association		0.1630	***0.0009***	0.1595	0.5689	0.2204

All associations are adjusted by age and BMI. Significant p-values (< 0,05) are marked in bold italics.

^a^PDEN: Percent Density.

^b^ABDEN: Absolute Density, measured in cm^2^.

Both haplotype variants identified in *IGFBP3 *were significantly associated with IGFBP3 plasma levels with a global p-value of 0.0009. Haplotype variant 1 was associated with lower IGFBP3 levels (p = 0.0009) whilst haplotype 2 was associated with increased IGFBP3 levels (p = 0.0008). After stratification by HT, these associations were still significant in the group that were never/past HT users (p = 0.004 and 0.003 for haplotypes 1 and 2 respectively) but not among current HT users (p = 0.3833 and 0.4220).

None of the common haplotypes within the *IGF1 receptor *(*IGF1R*) haplotype or *IGFALS *haplotype were found significantly associated with any of the parameters studied. There was a borderline association between *IGFALS *haplotype 1 and 3 and IGFBP3 levels (p = 0.0582 and 0.0769 respectively). After stratification by HT, haplotype 1 was significantly associated with the IGFBP3 level (p = 0.0309), while haplotype 3 was still borderline significant (p = 0.0639) in the never/past HT group.

### Stratified analysis by age and BMI

We examined if the significant association found between *IGF1 *haplotype 4 and absolute mammographic density was specific to groups of age or BMI, and stratified the analysis by tertiles of age and tertiles of BMI (see Additional file 3). Women within the age groups > = 59 to < 64, and > = 64 years (Tertile 2 and 3) carrying the haplotype 4 variant had a trend towards higher levels of mammographic density (p = 0.0976, p = 0.0879). Stratification by BMI, revealed a trend towards higher levels of mammographic density (p = 0.0989) for the women in BMI tertile 2.

### Single SNP analysis

In the six abovementioned genes we had 24 SNPs that were analyzed for association to the levels of IGF1, IGFBP3 and mammographic density (see Additional file 4). Eight of the 24 SNPs were found to be significantly associated with one or more of the parameters studied. In *IGF1R*, rs907806 was found to be significantly associated with the levels of IGFBP3 (p-trend = 0.0111). SNP rs3743259 was found to be significantly associated with mammographic density measured as both percent (p-trend = 0.0328) and absolute (p-trend = 0.0389) mammographic density. SNP rs2229765 and rs2016347 were significantly associated with mammographic density measured as both percent and absolute mammographic density, p-trend = 0.0265 and 0.0100, and, p-trend = 0.0434 and 0.0160 respectively. In *IGF2R*, rs998075 and rs998074 were found significantly associated with IGF1 (p-trend = 0.0072, p-trend = 0.0083) and IGFBP3 levels (p-trend = 0.0359, p-trend = 0.0320).

In *IGFALS*, rs9282731 was found significantly associated with the levels of IGFBP3 (p-trend = 0.0205). In *IGFBP3*, rs2471551 was found significantly associated with the levels of IGFBP3 (p-trend = 0.0009), denoting an association in cis.

## Discussion

This population-based cross-sectional study shows an association between a common genetic haplotype in *IGF1 *and absolute mammographic density in postmenopausal women after adjustment for age and BMI. Although not statistically significant, stratification by age and BMI revealed that the upper age tertiles and middle BMI tertile increased the mammographic density level. One haplotype in *IGF2 *was associated with the levels of both IGF1 and IGFBP3, while two haplotypes in the *IGF2R *gene were associated with the levels of IGF1. Within the *IGFBP3 *gene, two haplotypes were found associated with the IGFBP3 level indicating a regulation in cis.

The strength of our study is the large sample size and the fact that the samples were collected as part of a population-based screening project with high attendance rate[37]. Highly experienced personnel that were blinded to the characteristics of the women performed the reading and measurements of both the mammographic density and hormone levels. Also, we had information on age, BMI and HT use and were able to both adjust and stratify for these variables when necessary.

The limitation of our study is that the associations made with the polymorphisms within *IGF2R *were difficult to interpret due to the lack of measurements of its ligand IGF2. The women in the study were all postmenopausal, and some were taking HT, which could influence the circulating levels of IGF1 and IGFBP3. Furthermore, HT is demonstrated to have an impact on mammographic measurements, increasing the density. However, as HT cannot have influenced genotype, it is not technically a confounder, and as in most other analyses of mammographic density, we adjusted for age and BMI. We did however, stratify for HT, predominantly because of the possibility that HT use could be an effect modifier, i.e. have modified the effect of genotype on mammographic density.

Mammographic density is reduced by successive pregnancies and menopause, as well as with advancing age. Furthermore, mammographic density may reflect the cumulative exposure to hormones and growth factors that stimulate cell division and growth in the breast. Pike and colleagues proposed a model, stating that the effects of hormone exposure throughout life and the accumulation of genetic damage may cause an increased probability of breast cancer later in life [44]. The age-specific absolute risk of breast cancer caused by mammographic density is not yet determined, and it is unknown whether interventions that reduce cumulative exposure to density will reduce risk of breast cancer [2]. Because of the role of the IGF pathway in breast development and cellular proliferation, genetic variation within this pathway is of interest. Similar to mammographic density, IGF1 levels are related to age [22], BMI[45], and menopause status[21] and young women tend to have higher IGF1 levels than women in their postmenopausal years[22].

A positive association of IGF1 and IGFBP3 levels in relation to mammographic density in premenopausal women was found in most[17-20,32]but not all [46-48] previous studies. In postmenopausal women however the results are less consistent [18-21,47]. Data already published from this study were positive[21], suggesting an association between IGF1 and mammographic density. Our finding of a common haplotype in *IGF1 *associated with mammographic density is in agreement with previously published findings of an association with IGF1 levels and density. Associations of genetic variants in *IGF1 *and mammographic density in postmenopausal women can potentially better reflect the lifetime exposure of IGF1[11] compared to the IGF1 level measured at a certain time point, at a late stage, in a woman's life.

Other studies [18-20,46,47] such as the one of Dos Santos Silva *et al*. found no association between postmenopausal mammographic density and levels of IGF1, IGF2 or IGFBP3, nor the ratio of IGF1/IGFBP3, although, there was an association between the mammographic lucent area and IGFBP3 serum levels[47]. In summary, many studies have looked at variations in the IGF genes and their relationship with IGF plasma levels and mammographic density, but the results remain inconclusive, emphasizing the need for more studies.

The incidence of breast cancer has been associated with levels of IGF1 and IGFBP3 in premenopausal women in most [22,49-53], but not all studies [54,55]. Hankinson *et al*. performed a nested case-control study and found that IGF1 levels were higher among premenopausal women who developed breast cancer before age 50 than among age matched women who remained cancer free. For the postmenopausal women in the study no such association was established[22]. In postmenopausal women the findings are less clear and positive association of either IGF1, IGFBP3 or both with breast cancer [49,50,56] has been reported while other studies are negative[22,54,57]. The use of HT by postmenopausal women is known to lower both IGF1 and IGFBP3 levels significantly; thus the IGF1-associated increase in mammographic density seen in the non-HT users, may be difficult to observe in the HT users[21]. Nevertheless, one study reported an increased risk of breast cancer with increasing IGF1 levels also for postmenopausal HT-users (>55 years)[54]. Despite lowering the IGF1 levels, HT increases the mammographic density for most women and the age related decrease in mammographic density around the age of 55-64 does not commence in these women [58]. These findings support an emerging model of crosstalk between IGF1 and estrogens, suggesting that estrogens act through their receptor (ER) and affect the IGF1 expression[59].

### IGF1 haplotypes

Variation in mammographic density due to polymorphisms in the *IGF1 *gene has been reported in both pre- and postmenopausal women, but the association between genetic variants in *IGF1 *and mammographic density in breast tissue in postmenopausal women has been inconclusive [11,17,46].

Among the common haplotypes analyzed in *IGF1 *the least frequent haplotype was statistically significantly associated with an increase in ABDEN levels. This haplotype consists of the major allele of rs6220 and the minor allele of rs2162679. Other studies have reported an association between the minor allele of rs6220 and mammographic density in premenopausal women[17,60] but to our knowledge, no other study has looked at this SNP in relation to postmenopausal mammographic density. Separately, the SNPs comprising this haplotype have been reported to be associated with IGF1 levels and breast cancer risk. The polymorphism rs6220 has also been correlated with elevated IGF1 levels whereas homozygosity G/G of rs2162679, has been associated with reduced breast cancer risk as well as reduced levels of IGFBP3[25]. This is in agreement with our observation of increased mammographic density for haplotype 4, given that low levels of IGFBP3 and high levels of IGF1 have been reported to increase mammographic density[19].

It is surprising to find the *IGF1 *haplotype associated with increased mammographic density most strongly in women with higher BMI at an older age. However, since postmenopausal production of estrogens takes place predominantly in the adipose tissue, an increase in BMI would hypothetically result in increased estrogen levels. In turn estrogens may increase cellular IGF1 through crosstalk, and IGF1 may up-regulate the receptor response to estrogens[61]. This haplotype has a frequency of 0.04947 in the population studied which equal to 45 women and could be said to have little power. However, similar results have been reported by Muti *et al*. who reported that heavier postmenopausal women (BMI>26) had IGF1 levels associated with breast cancer risk [61]. Analysis of the SNPs in *IGF1 *did not reveal any significant associations with any of the parameters studied (IGF1, IGFBP3, IGFratio or mammographic density), and thus we were unable to verify the previous findings regarding these SNPs and association to IGF1 levels and breast cancer risk. However, this is an indication that the aforementioned association of the *IGF1 *haplotype 4 with mammographic density is dependent on the co-occurrence of these two SNPs.

### IGFBP3 haplotypes

Several associations of *IGFBP3 *polymorphisms and levels of IGFBP3 have been reported [25,46,47,62,63] one example is the -202(rs2854744) polymorphism associated with increased levels of IGFBP3 [46,47,62]. The -202 SNP has been associated with levels of IGF1 [60], IGFBP3 [46,47,60] and premenopausal mammographic density [46] but not with postmenopausal mammographic density [46,47]. The present study examined SNPs in the surrounding area of the -202 polymorphism which is an area suggested to be in strong LD[25,64].

The two *IGFBP3 *haplotypes analyzed here were found to be significantly associated with the levels of IGFBP3 suggesting a putative regulatory effect *in cis*. The C allele of SNP rs2471551 of the *IGFBP3 *haplotype has previously been associated with increased levels of IGFBP3 in combination with surrounding SNPs[25]. Upon single SNP analysis we found a significant trend of SNP rs2471551 with the level of IGFBP3 indicating that having two copies of the frequent allele C increase the least square mean of IGFBP3 compared to having two copies of the rare allele G, confirming the finding of Canzian *et al*. 2006. In the haplotype analysis we found that the AC haplotype was associated with higher levels of IGFBP3 than the AG haplotype and can thus confirm the result published by Canzian *et al*. that SNP rs2471551 is associated with increased IGFBP3 levels[25]. In addition, these haplotypes signify a trend of association with low levels (AG) and high levels (AC) of mammographic density (Table 3). These findings are consistent with the review of Fletcher *et al*. in which most reports agree on increased breast cancer risk with high levels of IGFBP3[65]. This may most strongly apply to premenopausal women, in whom the IGF levels are higher than in postmenopausal[22], and where the IGF axis is postulated to have an increased role because of involvement of the sex hormones[52]. The emerging belief that higher levels of IGFBP3 may decrease mammographic density and may decrease the risk of cancer[22,25,66] through low IGF1/IGFBP3 ratio must be further substantiated.

### IGF2 haplotypes

Even though it is known that the circulating IGF2 concentration is much higher than that of IGF1, there is limited evidence on its mitogenic activity in relation to breast cancer and disease[47,49,56], thus implications of IGF2 on breast cancer risk are inconclusive. Studies on genetic variants of *IGF2 *in relation to breast disease are few, and to our knowledge, the present study is the first to look at *IGF2 *polymorphisms in relation to levels of mammographic density, IGF1 and IGFBP3. The finding of a common haplotype (4, Table 3) significantly associated with higher levels of both IGF1 and IGFBP3 may be explained by a decrease in clearance of IGF1 due to potentially lower levels of IGF2. IGFBP3 is the principal carrier of both IGF1 and IGF2 and the possibility of a regulatory feedback of IGF1 and IGFBP3 through polymorphisms in *IGF2 *cannot be excluded. However, we did not have measurements of IGF2 and could therefore not test this. The association with haplotype 4 was no longer seen in analyses of the single *IGF2 *SNPs and thus there is reason to believe that the association is dependent on the combination of the two SNPs comprising this haplotype. After stratifying the analysis by HT the association was still significant for the women currently taking HT, implying that HT could be an effect modifier of this association. The sex hormones play an important role in the IGF axis and could be the reason why the association is stronger in these women.

### IGF1R haplotypes

The IGF1 receptor (IGF1R) sets off a complex cascade of signals upon binding of its ligands, IGF1 and IGF2[67]. IGF1R functions as an anti-apoptotic agent by enhancing cell survival, and has been found expressed in most breast cancer cell lines[68] and highly over-expressed in most malignant tissues[69]. In a study on genetic variation and breast cancer survival, Deming *et al*. [64] found SNP rs951715 within the *IGF1R *gene associated with breast cancer survival in postmenopausal women, whereas SNP rs2229765 included in the present study, was not[64]. SNP rs2229765 results in a silent mutation, and has thus far not been found associated with any epidemiological traits. In the haplotype analysis none of the *IGF1R *haplotypes were found significantly associated with any of the studied parameters. The single SNP analysis revealed significant association of SNP rs2229765 with both percent and absolute mammographic density, increased numbers of the G allele increased the least squares means of mammographic density. In addition the SNPs rs3743259 and rs2016347 were also significantly associated with percent and absolute mammographic density, and in both cases increased number of the most frequent allele increased the least square mean of mammographic density. Functional studies are needed to investigate if these SNPs influence the affinity to IGF1 and IGF2 increasing their growth promoting effects and possibly mammographic density.

### IGF2R haplotypes

IGF1 and IGF2 send their mitogenic and antiapoptotic signals through a common thyrosine kinase receptor, the IGF1R. Modulation of the mitogenic pathway occurs in part via the M6P/IGF2R, which functions in the internalization and degradation of IGF2[27]. IGF2R is also important in the activation process of TGFβ, which amongst other properties has the ability to inhibit cell growth. Loss of heterozygosity (LOH) of the M6P/IGF2R has been linked to liver and breast cancers, whereas somatic mutations of the M6P/IGF2R have been found in cancers of the prostate, lung, endometrium, brain, stomach and colorectum[27]. Chen *et al*. found that decreased ribosomal expression of the receptor leads to increased proliferation of MCF7 cells by a IGF2 related mechanism, mediated through IGF1R [27]. These findings have led to the suggestion that *IGF2R *is a tumor suppressor gene. Our results show that two of the *IGF2R *haplotypes are significantly associated to decreased levels of IGF1. For haplotype 1 the association was still significant after stratification for HT, in women that are never or past users of HT. Postmenopausal women that are not under the influence of hormones potentially have lower IGF1 levels. Analysis performed on the individual SNPs supports this finding, with a significant association of two *IGF2R *SNPs and the levels of IGF1, in addition they are also significantly associated with the levels of IGFBP3. IGF1 which is produced in the liver is influenced by several factors such as growth hormone and insulin, and its bioavailability is regulated by IGF2, IGFBPs and Als (acid-labile protein subunit)[65]. It is well known that IGF2 can act through IGF1R, in contrast IGF1 does not act through IGF2R, and to our knowledge no association between *IGF2R *and IGF1 levels have been described. IGF2R is able to degrade IGF2 and thereby regulates the circulating concentration of IGF2, in turn IGF2 clearance has the ability to regulate the level of IGF1. Thus, a possible explanation for the association of the two haplotypes and the SNP within *IGF2R *with IGF1 levels could be a change in clearance of IGF2 levels leading to decreased production of IGF1 through a regulative feedback loop. Whether or not such an interaction is present between IGF2 and IGF1 levels is impossible to confirm, due to lacking measurements of circulating IGF2.

### IGFALS haplotypes

Despite being an important member in IGF regulation, few studies have looked at this protein and variations within it in regards to breast cancer[25,64]. Canzian *et al*. [25] studied three SNPs within exon 2 of *IGFALS *in regards to breast cancer risk, two of which are included in the *IGFALS *haplotype of our study (rs3751893, rs17559), and found that homozygous carriers of SNP rs3751893 were associated with reduced circulating levels of IGF1. Deming *et al*. conducted a study on *IGFALS *promoter SNPs in relation to menopausal status but found no association [64].

The *IGFALS *haplotypes in this study were not significantly associated with neither the levels of IGF1, IGFBP3, their ratio nor mammographic density. Stratification by HT of the haplotype analysis revealed a significant association of the never/past HT group with the level of IGFBP3, in addition the SNP analysis revealed significant association of SNP rs9282731 with the level of IGFBP3. Increased number of the C allele increases the IGFBP3 level compared to the rare allele G. One hypothesis could be that the C allele modifies the IGFALS and reduces either its affinity or reduces its level causing increased level of free IGFBP3. Although the SNP analysis is based on low frequencies, functional studies could to be done to verify such a hypothesis. Further investigation into the role of this protein is needed to establish its involvement in the development of mammographic density and breast cancer.

## Conclusion

In conclusion, haplotypes were defined for each of the six genes from the *IGF *pathway studied here. Four genes had common haplotype variants (>5%) significantly associated with the metabolic levels of the gene products and mammographic density. One haplotype variant in *IGF1 *was found associated with mammographic density. In *IGF2 *one haplotype variant was associated with the level of both IGF1 and IGFBP3. Two haplotype variants and two SNPs in *IGF2R *were associated with the levels of IGF1. Both variants of the *IGFBP3 *haplotype and one SNP were associated with IGFBP3 level, indicating a regulatory function in *cis*.

## Abbreviations

SNP: Single Nucleotide Polymorphism; ABI: Applied Biosystems; HT: hormone therapy; GH: growth hormone; ALS: Acid Labile Subunit; TMBC: Tromsø Mammography and Breast Cancer study; ELISA: Enzyme-linked immunosorbent assay; IARC: International Agency for Research on Cancer; EDTA: ethylenediaminetetraacetic acid; DNA: deoxyribonucleic acid; UV: Ultra Violet light; NTC: No template control; EM: Expectation Maximization; LD: Linkage Disequilibrium; BMI: Body Mass Index; ABDEN: Absolute Breast Density; PDEN: Percent Breast Density.

## Competing interests

The authors declare that they have no competing interests.

## Authors' contributions

MB analyzed the results and wrote the manuscript, IB carried out the statistical analysis in SAS in collaboration with TF and ITG. FJ contributed to the statistical analysis in Haploview, HS contributed to the interpretation of the statistical analyses. GIGA performed the DNA isolation and sample preparation. YB was involved in the epidemiological part of the studies SC at the NCI. NIH provided laboratory analyses, where MY designed the assays and LB was instrumental for the genotyping. GU read the mammograms and supervised the statistical analysis, ITG is the principle investigator of the Tromsø Mammography and Breast Cancer Study and VNK is the principal investigator of the molecular part of the present study. VNK established the concept, designed and organized this study. All authors read and approved the manuscript.

## Pre-publication history

The pre-publication history for this paper can be accessed here:

http://www.biomedcentral.com/1755-8794/3/9/prepub

## Supplementary Material

Additional file 1 **Haplotype analysis stratified by current HT use**. Associations of the common haplotypes with IGF1, IGFBP3 and mammographic density levels stratified by current HT use. Click here for file

Additional file 2 **Haplotype analysis stratified by never/past HT use**. Associations of the common haplotypes with IGF1, IGFBP3 and mammographic density levels stratified by never past HT use. Click here for file

Additional file 3 **IGF haplotype association stratified by age and BMI**. Significant association of IGF1 haplotype 4 with ABDEN stratified by age and BMI tertiles. Click here for file

Additional file 4 **Associations of single SNPs with IGF1, IGFBP3 levels, IGFratio, and mammographic density**. PDEN-Percent Density, ABDEN-Absolute density. LSMEANS-Least squares Means. Measurements of mammographic density are boxcox transformed. Click here for file
